# Genome Sequences of Six *Paenibacillus larvae Siphoviridae* Phages

**DOI:** 10.1128/genomeA.00101-15

**Published:** 2015-06-18

**Authors:** Susan Carson, Emily Bruff, William DeFoor, Jacob Dums, Adam Groth, Taylor Hatfield, Aruna Iyer, Kalyani Joshi, Sarah McAdams, Devon Miles, Delanie Miller, Abdoullah Oufkir, Brinkley Raynor, Sara Riley, Shelby Roland, Horace Rozier, Sarah Talley, Eric S. Miller

**Affiliations:** a Department of Plant & Microbial Biology, North Carolina State University, Raleigh, North Carolina, USA; b Phage Hunters and Phage Genomics first-year courses and Biotechnology Program, NC State University, Raleigh, North Carolina, USA

## Abstract

Six sequenced and annotated genomes of *Paenibacillus larvae* phages isolated from the combs of American foulbrood-diseased beehives are 37 to 45 kbp and have approximately 42% G+C content and 60 to 74 protein-coding genes. Phage Lily is most divergent from Diva, Rani, Redbud, Shelly, and Sitara.

## GENOME ANNOUNCEMENT

Phages were isolated from infected comb presented to the North Carolina Department of Agriculture from across the state. Phage isolation and propagation used *Paenibacillus larvae* host strains ATCC 9545 and ATCC 25747 grown on brain heart infusion (BHI) plus 0.4% glucose and 1 µg/ml thiamine in broth or agar at 30°C. Swabs were taken from infected comb cells and incubated in growth medium with selective bacteria for 24 to 48 h. Plaques were identified on top agar lawns of *P. larvae* and reisolated from plate streaks at least three times. Electron micrographs showed that all six phages have the *Siphoviridae* morphotype.

Confluent lysis on *P. larvae*-seeded top agar was used to prepare lysates from which DNA was extracted using the Promega DNA cleanup system. Genomic DNA was sequenced in the North Carolina State University (NCSU) Genomic Sciences Laboratory using fragmentation, sizing, adapter addition, and flow cell usage, as described (Illumina, Inc., San Diego, CA). Lily and Rani were sequenced by HiSeq 100-bp reads, with the remaining four genomes being sequenced using MiSeq 2 × 300-bp reads. CLC Genomics Workbench releases 2013 and 2014 were used for assembly. The physical ends of the Lily and Rani genomes were determined by ligation of genomic DNA, PCR amplification across the joined ends, and Sanger sequencing. All use cos packaging and are likely temperate phages. Lily has a 12-bp 5′ overhanging terminus (GGTGCGCGTGAG), and Rani has a 9-bp 3′ overhanging terminus (CGACTGCCC). Diva, Redbud, Shelly, and Sitara have physical genome ends like those of Rani, based on matching end nucleotides and similar sequence assembly patterns.

The genomes were annotated by students using DNA Master (http://cobamide2.bio.pitt.edu) on the NC State Virtual Computing Lab. Coding regions were predicted using Glimmer (1) and GeneMark (2), and start codons were chosen based on DNA Master ribosomal binding site (RBS) parameters. The absence of tRNAs was predicted using ARAGORN (3) and tRNAscan (4). Protein function and start sites were corroborated using NCBI BLASTp (5).

All six genomes encode two terminase subunits oriented at the 5′ end of the sequence, followed by genes for the tail and head that are generally syntenic. Rani and Redbud are nearly identical, and Lily is the most divergent. All contain genes typically seen in *Siphoviridae* phages (major capsid, portal, tape measure, tail, holin, endolysin, etc.). Most of the phages share regions of similarity with phiIBB_Pl23, a *P. larvae Siphoviridae* phage (6), and negligible similarity with the *Myoviridae* phages isolated from Utah (7). That all of the reported phages from Utah are *Myoviridae* and the North Carolina phages are *Siphoviridae* may reflect that the North Carolina phages were uniquely isolated from American foulbrood (AFB) diseased hives or that the Utah host used is substantially different from that of the *P. larvae* strains used in this work. Phage HB10c2 (GenBank accession no. KP202972), isolated from an AFB-diseased hive in Germany, has substantial sequence similarity to the genomes reported here.

### Nucleotide sequence accession numbers. 

The nucleotide sequence accession numbers are listed in Table 1.

**TABLE 1:** Characteristics of the phages in this study

Phage	GenBank accession no.	Strain host	Coverage (×)	Length (bp)	G+C content (%)	No. of genes
Diva	KP296791	ATCC 9545	18,260	37,246	42.1	60
Lily	KP296792	ATCC 9545	30,000	44,952	42.7	74
Rani	KP296793	ATCC 9545	33,000	37,990	41.8	61
Redbud	KP296794	ATCC 9545	14,801	37,971	41.8	61
Shelly	KP296795	ATCC 9545	7,815	41,152	41.5	68
Sitara	KP296796	ATCC 25747	6,516	43,724	41.6	74

## References

[1] Delcher AL, Harmon D, Kasif S, White O, Salzberg SL 1999 Improved microbial gene identification with Glimmer. Nucleic Acids Res 27:4636–4641. doi:10.1093/nar/27.23.4636. 10556321 PMC148753

[2] Besemer J, Lomsadze A, Borodovsky M 2001 GeneMarkS: a self-training method for prediction of gene starts in microbial genomes. Implications for finding sequence motifs in regulatory regions. Nucleic Acids Res 29:2607–2618. doi:10.1093/nar/29.12.2607. 11410670 PMC55746

[3] Schattner P, Brooks AN, Lowe TM 2005 The tRNAscan-SE, snoscan and snoGPS Web servers for the detection of tRNAs and snoRNAs. Nucleic Acids Res 33:W686–W689. doi:10.1093/nar/gki366. 15980563 PMC1160127

[4] Laslett D, Canback B 2004 ARAGORN, a program to detect tRNA genes and tmRNA genes in nucleotide sequences. Nucleic Acids Res 32:11–16. doi:10.1093/nar/gkh152. 14704338 PMC373265

[5] Altschul SF, Gish W, Miller W, Myers EW, Lipman DJ 1990 Basic local alignment search tool. J Mol Biol 215:403–410. 2231712 10.1016/S0022-2836(05)80360-2

[6] Oliveira A, Melo LD, Kropinski AM, Azeredo J 2013 Complete genome sequence of the broad-host-range *Paenibacillus* larvae phage phiIBB_Pl23. Genome Announc 1(5):e00438-13. doi:10.1128/genomeA.00438-13. 24009112 PMC3764407

[7] Sheflo MA, Gardner AV, Merrill BD, Fisher JN, Lunt BL, Breakwell DP, Grose JH, Burnett SH 2013 Complete genome sequences of five *Paenibacillus larvae* bacteriophages. Genome Announc 1(6):e00668-13. doi:10.1128/genomeA.00668-13. 24233582 PMC3828306

